# Systematic Identification and Characterization of RNA Editing in Prostate Tumors

**DOI:** 10.1371/journal.pone.0101431

**Published:** 2014-07-18

**Authors:** Fan Mo, Alexander W. Wyatt, Yue Sun, Sonal Brahmbhatt, Brian J. McConeghy, Chunxiao Wu, Yuzhuo Wang, Martin E. Gleave, Stanislav V. Volik, Colin C. Collins

**Affiliations:** 1 Vancouver Prostate Centre & Department of Urologic Sciences, University of British Columbia, Vancouver, BC, Canada; 2 Department of Experimental Therapeutics, BC Cancer Agency, Vancouver, BC, Canada; University of Florida, United States of America

## Abstract

RNA editing modifies the sequence of primary transcripts, potentially resulting in profound effects to RNA structure and protein-coding sequence. Recent analyses of RNA sequence data are beginning to provide insights into the distribution of RNA editing across the entire transcriptome, but there are few published matched whole genome and transcriptome sequence datasets, and designing accurate bioinformatics methodology has proven highly challenging. To further characterize the RNA editome, we analyzed 16 paired DNA-RNA sequence libraries from prostate tumor specimens, employing a comprehensive strategy to rescue low coverage sites and minimize false positives. We identified over a hundred thousand putative RNA editing events, a third of which were recurrent in two or more samples, and systematically characterized their type and distribution across the genome. Within genes the majority of events affect non-coding regions such as introns and untranslated regions (UTRs), but 546 genes had RNA editing events predicted to result in deleterious amino acid alterations. Finally, we report a potential association between RNA editing of microRNA binding sites within 3′ UTRs and increased transcript expression. These results provide a systematic characterization of the landscape of RNA editing in low coverage sequence data from prostate tumor specimens. We demonstrate further evidence for RNA editing as an important regulatory mechanism and suggest that the RNA editome should be further studied in cancer.

## Introduction

The deregulation of post-transcriptional modification is increasingly recognized as a hallmark of cancer, generating enormous diversity and significantly affecting downstream activity. RNA editing is a process by which the sequence of primary transcripts is modified, resulting in RNA-DNA sequence differences (RDDs). The most common type of RNA editing results from action of the adenosine deaminase acting on RNA (ADAR) class of enzymes, which catalyze the conversion of adenosine (A) to inosine (I) in double stranded RNA [1]. A→I editing is highly prevalent within inverted-repeated Alu elements due to their propensity to form double-stranded RNA structures [2],[3]. However, RNA editing also affects introns and untranslated regions (UTRs) of genic transcripts (partly due to the presence of Alu elements within these regions), where substitutions can modulate splicing or RNA structure [4], [5]. Indeed, a recent study reported that RDDs were enriched in 3′UTRs and microRNA target sites in mouse tissues, suggestive of a regulatory role for RNA editing [6]. Moreover, a database of predicted A→I editing miRNA binding sites has been built[7]. Furthermore, since inosine base pairs with cytidine, and is interpreted by the translational machinery of a cell as guanine, RNA editing can cause non-synonymous changes to coding regions, although to date only a few genes have proven to be recurrently altered in this manner [8], [9]. The most notable recurrently edited site falls within the second transmembrane domain of mammalian glutamate receptor subunits, where it results in a Q to R substitution, thereby controlling calcium permeability[10].

The advent of whole transcriptome sequencing has permitted systematic discovery of RNA edits, and huge numbers of putative RNA editing sites are being reported across the genome [11], [12], [13]. Unfortunately, designing accurate bioinformatics methodology with low false positive rates has been fraught with challenges since true RNA editing events are difficult to distinguish from sequence or mapping errors, or even DNA polymorphisms and somatic mutations.

Prostate cancer is a leading cause of cancer-related death [14]. Recent whole genome and transcriptome sequencing studies of hundreds of prostate tumors have defined novel molecular subtypes and characterized extensive genomic aberration underlying disease initiation and progression [15], [16], [17]. RNA editing deregulation has begun to be linked to cancer, including in hepatocellular carcinoma, where recurrent editing of *AZIN1* promotes pathogenesis [8], [18], [19]. However, there have been no reports to date in prostate cancer. Here, we present analysis of 16 paired DNA-RNA sequence libraries from prostate tumor specimens, employing a comprehensive strategy to rescue low coverage sites and minimize false positives. We identified thousands of recurrent putative RNA editing sites across transcriptome, including hundreds predicted to result in deleterious amino acid alterations. Finally, we report a potential link between RNA editing of microRNA binding sites and up-regulation of the edited transcripts. Overall our results provide a systematic and unbiased characterization of RNA editing features in low coverage sequence data from prostate tumor specimens. We demonstrate further evidence for RNA editing as a regulatory mechanism and suggest that the RNA editome should be further studied as a mutational mechanism in prostate cancer.

## Results

### Rescue of RNA-DNA different (RDD) sites with low DNA coverage

**Figure 1:**
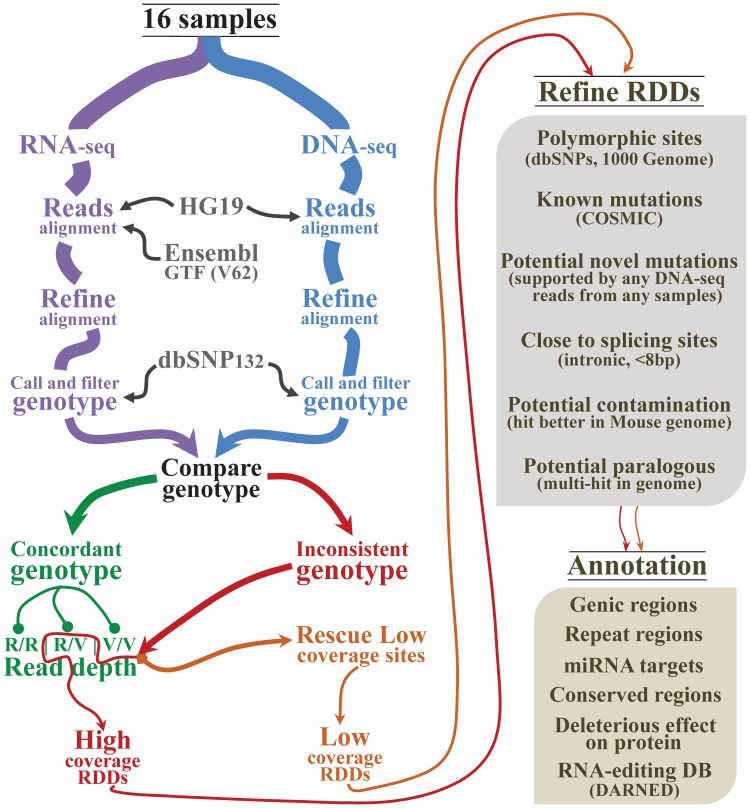
The computational workflow employed to detect RDDs. The thickness of the arrowed lines loosely illustrates the number of candidate RDDs passing each filter.

**Figure 2:**
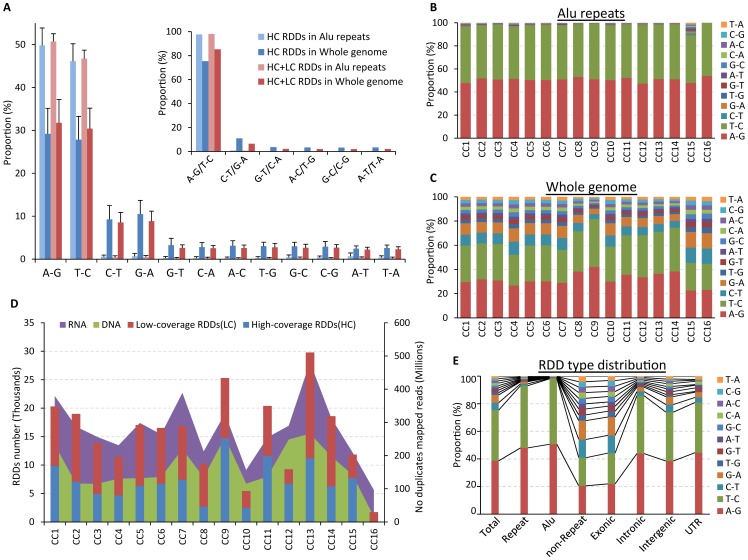
The distribution of RNA-DNA differences across the prostate cancer genome. A) RDD site type, whether high-coverage (HC) or high and rescued low coverage (LC) combined sites in Alu regions or across the whole genome showing the pervasive nature of A→I (A→G or T→C) edits. B) Distribution of RDD type in Alu repeats for each sample. C) Distribution of RDD type across whole genome for each sample. Note the increase in the proportion of C→T (G→A) sites in CC15 and CC16. D) The number of mapped DNA/RNA reads after duplicate removal (stacked areas in green and purple) in each sample plotted together with the number of high/low coverage RDDs (stacked columns in blue and red) predicted, demonstrating a broad correlation between read depth and the number of RDD predictions. E) RDD site type distribution across the genome showing that C→T (G→A) sites are more common in non-repeats and exonic regions, potentially mediated by members of APOBEC family of RNA editing enzymes.

Using an in-house bioinformatics pipeline (Methods; Figure 1), we predicted RNA-DNA differences (RDD) in a cohort of published matched whole genome and transcriptome sequencing data from 16 prostatic cancer specimens (9 different patients and 2 cell lines; Table S1 in File S2). There are few studies which publish matched whole genome and transcriptome sequence data and as such we were restricted to using a disparate cohort comprised of different prostate tumor sub-types. Nevertheless, the detection of putative RNA editing events in next-generation sequence data is an emerging area of research, and we hypothesized that a deep systematic analysis of RDD sites (even in a limited sequence cohort) would provide novel insights into global RNA editing of the human transcriptome and guide future studies. We employed a stringent filtering strategy to minimize false positives (see Methods), excluding predicted RDDs which were: i) known polymorphisms or mutations; ii) supported by any DNA-seq read from any specimen; iii) mapped to within 8 bp of splice sites; iv) better explained by murine contamination; and v) within regions aligning to paralogous genes or repeats. After filtering we predicted a total of 109,690 RDDs, 56,114 which were low coverage sites salvaged by our rescue strategy (see Methods) necessitated by the low coverage (∼4X) of DNA-seq data. Previous studies have demonstrated that the most common type of RNA-editing event is A→I, mediated by ADAR [20], which is especially pervasive in Alu repeats (89.3% to 97.5% of sites) [12], [13], [14], [15], [16], [17], [21], [22], [23]. Therefore, to evaluate the suitability of including low coverage RDD sites in downstream analysis we considered the type of RNA edit predicted at these sites. We divided genomic regions into 3 categories: Alu repeats, non-Alu repeats and non-repeat regions. Among high coverage RDDs sites (i.e. those which reached the average sequencing depth of concordant genotype sites) in Alu repeats, 97.69% were A→G (or T→C, since we performed non-strand specific RNA sequencing). However, upon inclusion of rescued low coverage sites, the detection of A→G (T→C) RDDs in Alu repeats was slightly increased (98.09%), and the ratio of A→G (T→C) sites across the genome improved from 69.59% to 75.37% (Figure 2A-C; Table S2 in File S2). If we assume that all non-A→G (T→C) sites in Alu repeats reflect false positives, then the FDR (false discovery rate) of our prediction is 1.9%. As a final check, we simulated our rescue strategy using a second sequence dataset of matched DNA-RNA from the LNCaP cell line which has higher coverage of DNA-seq (∼27X) and RNA-seq (∼33X) (Table S1 in File S2). We down-sampled the dataset, removing 30%, 50% or 70% of the reads, before applying the same criterion of read depth as for the whole dataset to call and refine RDD sites. From 30% down-sampling, we still detected 91.62% of the high-coverage RDD sites originally called from the whole dataset. Moreover, 71.28% of these were salvaged by our rescue strategy. When we down-sampled by 50% and 70%, 77.68% and 56.14% of the original high-coverage RDD sites were detected, and all rescued by our method (Figure S1 in File S1). Additionally, all there were no ‘new’ RDD sites detected in down-sampling simulations that were not detected in the entire dataset, suggesting that the rescue strategy does not generate new false positives. Moreover, the RDDs validation success rate (by Sanger sequencing; described below) did not differ substantially between high and low coverage RDD sites (88% and 70% respectively). Thus we concluded that our rescued low coverage RDD sites merited continued consideration.

### The types of RDD sites identified

Although the overall number of RDDs predicted in each sample was tightly linked to sequencing depth (R^2^ = 0.86; Figure 2D), the distribution of RDD types was largely invariable between samples, regardless of genomic location (R^2^ ranging from 0.91 to 1) (Figure 2E, Figure S2 in File S1 and Table S3 in File S2).

**Figure 3:**
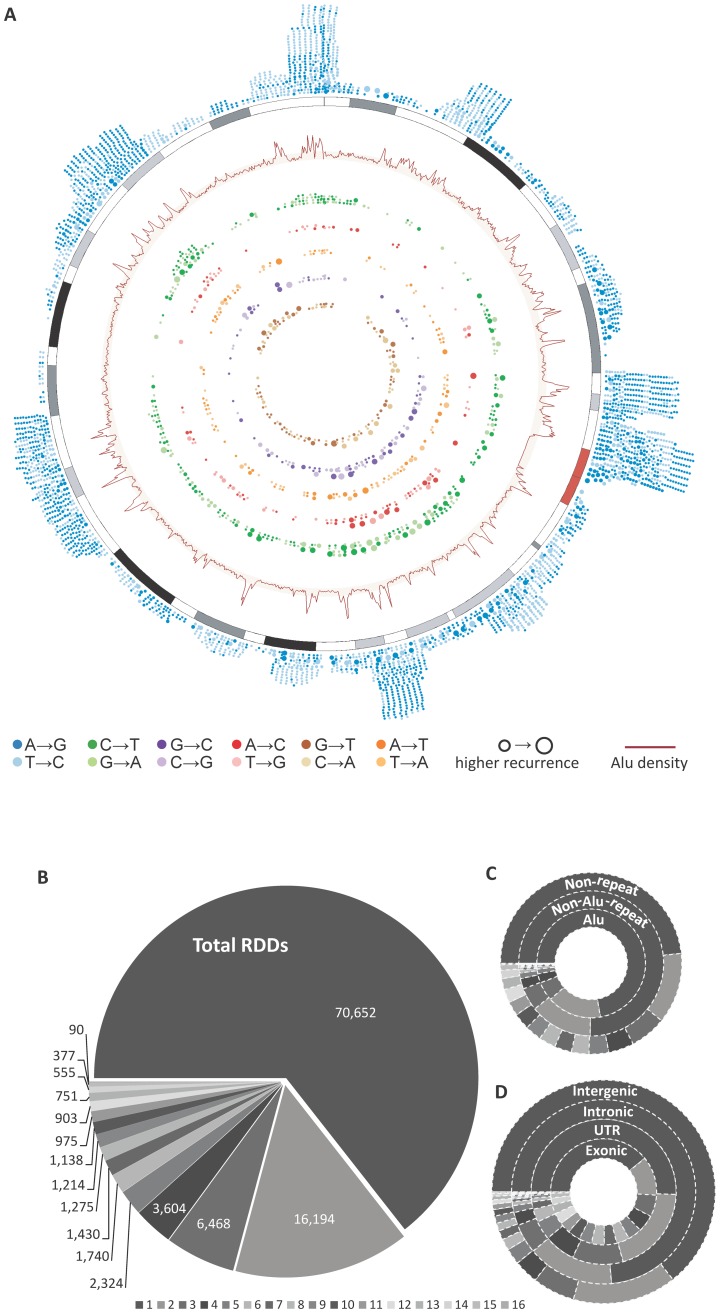
RDD density and frequency distribution. A) Circos plot depicting the landscape of RNA editing in a representative chromosome (chr8) across the cohort. The relationship between Alu element density (brown line) and A→G (or T→C) RNA editing events (blue dots) is clear. B) Recurrence distribution of all RDDs across the cohort, e.g. 70,652 were unique to one sample, 16,194 in two samples etc. C-D) Recurrence distribution of recurrent RDDs in different regions of the genome.

Globally, the majority of all RDDs (72,398 [65.63%]) fell in repeat regions, 84.46% of which were in Alu repeats (Figure 3A). In non-Alu repeat regions, A→G (T→C) sites were less pervasive (66.04%), giving way to more C→T (G→A) sites (14.63%), potentially due to the activity of members of the APOBEC enzyme family[24], [25] (Figure 2E; Figure S3 in File S1). This trend was more overt in non-repeat regions, with the proportion of A→G (T→C) sites falling to 40.91% and C→T (G→A) sites increasing to 26.86%. Although non-canonical substitution types were much less abundant (e.g. 0.15% of RDD sites in Alu repeats), they were more prevalent in non-repeat regions (Table S2 in File S2; Figure S2C in File S1).

We compared our RDD sites with the database of RNA editing (DARNED) [26]. Although only 4.14% of our A→G (T→C) RDD sites across the whole genome were also represented in DARNED (8.46% of the DARNED database), those sites were more likely to be recurrent (P<0.0001; Fishers exact test). However, when considering commonly edited genes (including exonic, UTR and intronic regions), rather than specific RDD sites, 8.68% (875/10,081) of the genes with A→G (T→C) RDDs were edited in DARNED (out of 2,390 genes) (Table S4 in File S2). Despite this increase in enrichment at the gene level, the DARNED database is clearly not yet fully comprehensive.

### RDDs are enriched in conserved regions and more likely to be recurrent

The majority of RDDs (64.41%) were unique to a single specimen (Figure 3B), but this ratio varied according to location, with repeat regions exhibiting the highest abundance of unique RDDs (72.28% and 75.22% of RDDs in Alu and non-Alu repeats respectively). Conversely, only 48.24% of RDDs in non-repeat regions were unique (Figure 3C). Furthermore, within genic regions the exons were most likely to harbor recurrent RDDs (>60% of RDDs in exons), followed by UTRs and introns (Figure 3D). These exonic recurrent sites may therefore represent functionally conserved elements or motifs, while the UTR and intronic RDDs may be partly driven by Alu elements within those regions (Table S2 in File S2). RDDs were generally enriched in conserved regions (Table S5 in File S2), with 18.01% (19,758) of total RDDs located in 9,684 conserved elements predicted by phastCons algorithm [27]. These RDDs included 63.03% and 72.13% of all detected coding RDDs and ncRNA RDDs respectively. The frequency of RDDs within conserved regions was 8,529.4 RDDs per Mb; significantly higher than the RDD rate across all edited transcript regions (1,005.6 RDDs per Mb). It is possible that RDDs play an important role in post-transcriptional regulation through altering conserved functional regions. Alternatively, although we stringently filtered out all RDDs mapping to paralogous genes or to the mouse genome contamination, we cannot rule out the possibility that some of these sites, especially non-canonical sites, are false positives caused by mapping errors [20]. However, it is important to note that if we considered only high coverage RDD sites the percentage of recurrent RDDs was ∼35%, similar to the frequency of high and low coverage RDDs combined, further indicating the usability of low coverage sites.

### A→G sites exhibited pronounced clustering

Consistent with previous reports [23], a large proportion of RDDs (46,875 [42.73%]) fell in clusters (see Methods) especially in ncRNAs (59.57%) and UTRs (57.61%) (Table S6 in File S2). A→G (T→C) sites in particular fell in clusters, accounting for 81.23% of all clustered sites (when A→G (T→C) sites account for only 75.37% of all RDDs). Over 90% of clusters involved exclusively A→G (T→C) RDDs, which would be consistent with regions of dsRNA being resolved by ADAR [28].

### The distribution of RDDs within genic regions

Across the cohort 12,642 genes were affected by RDDs within exonic, intronic, UTR or non-coding RNA regions. Most RDD sites (63.05%; range 60.1%-77%) resided within intronic and non-coding RNA regions, potentially related to enrichment of Alu elements (Table S7 in File S2; Figure S4 in File S1). However, the relative proportions of intronic RDDs and ncRNAs varied considerably, presumably due to overlapping annotations as many ncRNAs fall within introns (81% of all spliced human protein-coding genes have transcriptionally active introns [29]). It is well-established that both introns and ncRNAs exhibit secondary structure [30], [31], potentially enabling the activity of RNA editing enzymes. Nevertheless, on average over 14% of RDDs fall in ncRNAs. Conversely, when one considers SNVs supported in both DNA and RNA, only 1.3% fall in ncRNAs (introns 48.9% vs 64.5%). It is therefore conceivable that RNA editing is a far more relevant mechanism of plasticity in ncRNAs than SNVs.

In mature mRNAs (i.e. coding regions and UTRs), half (46.3%) of RDDs reside in 3′UTRs that frequently contain highly conserved elements targeted by miRNAs or RNA-binding proteins [32], [33]. This enrichment supports previous observations that RNA editing plays an important role in transcript expression regulation through creating or interrupting functional motifs [34].

4.08% of total RDDs reside within coding regions (Table S7 in File S2; range from 0.98% to 8.1% in individual samples), half of which are amino acid altering (non-synonymous). This is comparable to paired DNA-RNA SNVs in this dataset where 50% of coding mutations were non-synonymous. CC13 was an exception, with 8.1% of RDDs falling within coding regions, but only 18.7% of those were non-synonymous RDDs. Overall, only 34.9% of sites in coding regions were A→G (T→C), another 30.5% were C→T (G→A) (Table S2 in File S2) which is known to be more frequent in coding regions, partly due to higher GC content and frequency of CpG methylation.

Considering genes which were recurrently edited, regardless of specific RDD site, there were 2,898 genes with RNA editing to exonic regions detected in ≥2 samples (Table S8 in File S2). Furthermore, we noted that the correlation of RDD frequency within genes between pairs of samples was much stronger when comparing related samples (e.g. from the same patient or tissue) to unrelated: outperforming the same analysis using gene expression (Table S9 in File S2). RDD detection is biased by gene expression, but this data suggests that the RDDs we detected were broadly tissue or patient specific.

1552 RDDs in 910 genes were predicted to result in non-synonymous changes (including stop-codon gain or loss) to peptide sequences, and 369 of these genes (566 RDD sites) were recurrent (Table S8 in File S2). 546 genes had predicted ‘deleterious’ substitutions (Table S10 in File S2), including *GRIK1*, a kainite glutamate receptor, which harbored the well-studied Q to R substitution in the second transmembrane domain (Table S11 in File S2). Other examples included an RDD in the non-receptor tyrosine-protein kinase *ABL2* which was predicted to substitute a highly conserved S for a Y within the protein kinase domain. Additionally, 33% (180/546) of genes with deleterious substitutions of RNA editing have been reported to be mutated in several previous studies of prostate cancer [15], [16], [17], [35], suggesting a relative enrichment (Fisher's exact test P = 0.0146). Furthermore, the most enriched ‘Bio Functions’ from Ingenuity Pathway Analysis of the 546 genes with deleterious substitutions were ‘Cellular Movement’ and ‘Adenocarcinoma’, suggesting that some of the non-synonymous events could be disease-relevant.

### An association between RDDs in miRNA target regions and increased transcript expression

**Figure 4:**
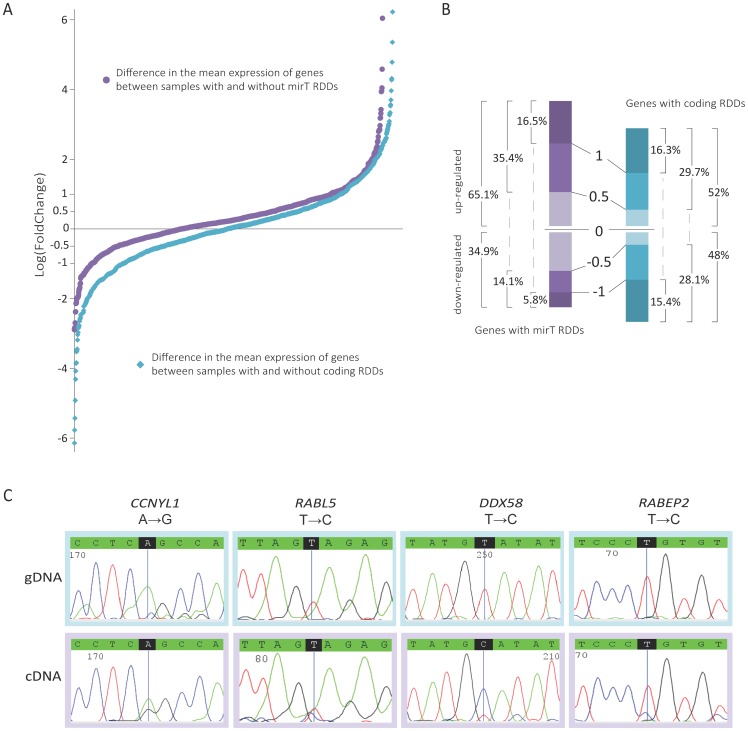
Association of microRNA target RDDs with gene expression. A) Distribution of the difference in expression of genes between samples with and without RDDs (log[FoldChange]). The proportion of up-regulated genes is higher when considering transcripts affected by miRNA-target RDDs (purple dots) compared to the proportion of genes up-regulated when affected by coding region RDDs (blue dots). B) Bar chart depicting the number of up/down-regulated genes when affected by miRNA-target RDDs (left) or coding region RDDs (right). The differentially graded blocks in each bar represent the number of genes under different cutoffs of log(FoldChange) (1, 0.5, 0, -0.5, -1), with the numbers besides bars indicating the proportion of genes under the cutoffs. For example, in genes with mirT RDDs (purple bar), 65.1% of genes were up-regulated with logFC >0 while 34.9% of genes were down-regulated with logFC <0; and 35.4% of genes were up-regulated with logFC >0.5 while 14.1% of genes were down-regulated with logFC <-0.5. C) Representative examples of Sanger sequencing validations of miRNA-target RDDs in the genes *CCNYL1*, *DDX58*, *RABL5* and *RABEP2* (complete list in Table S11 in File S2).

We hypothesized that RDDs in miRNA target regions (mirT RDDs) could affect transcript expression through avoidance of miRNA mediated regulation. In our dataset 3,023 RDDs were located within 6,451 miRNA target regions (1,027 miRNAs and 1,334 genes). 619 (46.4%) affected genes were unique to individual samples, but 140 (10.49%) were observed in more than half of the cohort (Table S12, S13 in File S2). We compared transcript expression of protein coding genes with and without mirT RDDs, but aware that our original ability to identify RDDs was influenced by transcript expression, we included only transcripts where sequencing coverage was greater than 10X. Of the 1,334 genes affected by mirT RDDs, 1,068 had evaluable expression. Remarkably, we observed that 65.1% of these genes had higher expression when affected by a mirT RDD (Table S14 in File S2; Figure 4A-B), rising to almost 75% of genes when considering only those with a logFC >1 in either direction. Furthermore, comparing only genes that had more than 2 specimens in each group (i.e. with or without mirT RDDs), revealed that 114 genes had significant upregulation when affected by a mirT RDD compared to just 1 gene that was significantly down-regulated (p<0.05; t-test). As a negative control we carried out the same analyses for coding region RDDs (crRDDs). We found that 52% (592/1,139) of genes had higher expression when affected by crRDDs: a result approaching random selection, and one that did not change when we considered only those genes with logFC >0.5 or 1 in either direction (Figure 4B). Overall therefore, the proportion of up-regulated genes with mirT RDDs was significantly higher than that of up-regulated genes with exonic RDDs (Fisher's Exact Test p = 2.7E-10), potentially suggesting that altering miRNA target regions through RNA editing has a positive effect on transcript abundance.

Since archival clinical material was extremely limited (and entirely absent for several specimens) we focused validation efforts on mirT RDDs identified in up-regulated genes with potential links to cancer. By PCR amplification of gDNA/cDNA and Sanger sequencing, we examined all mirT RDDs in those genes that were picked purely based on potential biological impact. Although validation of RNA editing events is notoriously difficult given their often transient nature and abundance variation as well as the insensitivity of Sanger sequencing of cDNA for low-level editing, we successfully validated 18/25 (72%) RDD sites, including those in *CCNYL1*, *DDX58*, *POLH*, *ZYG11A*, *RABL5* and *RABEP2* (Figure 4C, Table S11 in File S2). *RABL5* is a putative member of the *RAS* oncogene family, and *RABL5* up-regulation in a large clinical cohort of prostate tumors was associated with poor survival (p = 0.028) [36]. *DDX58* is a putative RNA helicase implicated in RNA binding and alteration of RNA secondary structure. *CCNYL1* is a crucial regulator of cell cycle transitions, and is up-regulated in prostate cancer [37], [38], [39], [40], [41], [42]. Furthermore, the four validated mirT RDDs in *DDX58* and *CCNYL1* resided in binding regions of miRNAs hsa-miR-10b, hsa-miR-98 and hsa-miR-122, which have all been reported to be dysregulated in prostate cancer relative to adjacent benign[43]. Other notable genes with mirT RDDs included *KIAA1324* and *ANAPC16*. The estrogen regulated gene *KIAA1324* (*EIG121*) is expressed >4X higher in the 8 specimens that exhibit mirT RDDs than those that did not. *KIAA1324* (*EIG121*) regulates autophagy and promotes cell survival under stress, and increased expression is associated with poor prognosis [44], [45]. *ANAPC16* is a component of the anaphase promoting complex/cyclosome (APC/C), a cell cycle-regulated E3 ubiquitin ligase that controls progression through mitosis and the G1 phase of the cell cycle.

To further confirm the reliability of our RNA editing discovery pipeline, we performed a second round of PCR validation in an independent set of 15 additional prostate cancer samples, including three tumors of Gleason score 6, two samples each from Gleason score 7, 8, 9, and 10, and 4 matched adjacent benign samples. We assessed matched cDNA and gDNA from these samples for the presence of the 18 different mirT RDDs validated above. Aside from a single RDD site in the gene *ZYG11A*, all mirT RDDs were successfully detected in this second cohort, with RNA editing ratios ranging from 5% to 95% by visual estimation of Sanger sequencing trace (Table S15 in File S2). There was no strong association between tumor Gleason grade and RNA editing ratios, although tumor samples of Gleason score 9 and 10 showed higher RNA editing ratios on average. Within the four matched tumor-benign pairs examined, two had a higher average RNA editing ratio in the tumor sample compared to the matched benign tissue (11% and 14% higher respectively) while the other two pairs showed a similar RNA editing level and a decrease of RNA editing ratio respectively. It is noteworthy that four samples (one of Gleason score 6, two of Gleason score 7 and one of Gleason score 8) showed very low RNA editing ratios, which may suggest that RNA editing is not highly active in some tumors, potential representing another layer of inter-tumor heterogeneity.

By examining the expression of genes with mirT RDDs in six published microarray datasets [37], [38], [39], [40], [41], [42], we found that 54.78% (ranging from 51.02% to 60.03%) up-regulated genes with mirT RDDs were also up-regulated in primary PCa relative to benign samples. This ratio dropped to 50.46% (ranging from 45.05% to 55.33%) when overlapping down-regulated genes with mirT RDDs with up-regulated genes in microarray datasets (Table S16 in File S2, Figure 5 in File S1). Although this difference is not significant, the fact that the same trend was present in each of the six microarray datasets is noteworthy. Pathway analysis on all up-regulated genes with mirT RDDs (logFC >0.5), implicated ‘Cancer’ as the most enriched ‘Bio Function’.

Finally, using the LNCaP cell line, we validated further 10 RDDs (out of 12 sites randomly selected; validation rate 83%) residing in protein-coding or non-coding transcripts (Table S11 in File S2). Combining these sites together with those described above revealed that the overall validation rate did not differ substantially between high and low coverage RDD sites (88% and 70% respectively). Furthermore, the *GRIK1* exonic site, which was also validated, was a low coverage RDD (Table S11 in File S2).

## Discussion

This is the first comprehensive evaluation and characterization of RNA editing using prostate tumor specimens, reporting over a hundred thousand putative RNA editing events, more than a third of which were recurrent. The challenge of low DNA sequencing coverage and extremely limited validation material demanded a stringent rescue strategy for RDD site identification, which is likely to be applicable to other datasets. The prediction of RNA editing events from transcriptome sequencing data is an emerging field, and there is no consensus methodology for analyses. This is confounded by the fact that most sequence datasets are not generated with RNA editing analyses explicitly in mind, and therefore do not feature ideal sequence depth and library quality, or the number of biological replicates, sufficient for optimizing accurate interrogation of the RNA editome [46]. Furthermore, there is also significant debate surrounding exactly what results to expect, in terms of the nature of base substitutions and the distribution and recurrence of RNA editing across the genome. In this context our results demonstrate that it is possible to use low coverage sequence data from a cohort designed for a completely different hypothesis, yet still generate insights to help guide future studies. Given the increasingly recognized importance of low abundance cell populations (e.g. cancer stems cells) in heterogeneous tumors our low coverage rescue strategy may have applicability beyond shallow sequence datasets. It is also worth noting that although our cohort is clearly limited, to our knowledge it still represents the largest number of cancer samples subjected to a comprehensive RNA editing analysis to date.

Emergent predictions were of high confidence as evidenced by: the proportion of canonical edits predicted; the high correlation of edited genes between related samples; the *de novo* detection of known RNA editing events (e.g. in *GRIK1*); and our PCR-based validations. Our data demonstrated a potential link between RNA editing of microRNA target regions and increased gene expression. It is possible that RNA editing is directly responsible for this increase in transcript abundance, by preventing binding of microRNAs and thereby causing the transcript to escape microRNA-mediated regulation. Although the differential expression of specific transcripts was not drastic, this is consistent with reports suggesting the effect of miRNA on transcript destabilization is at low-to-moderate levels[47], [48].

The sequencing of over 200 prostate cancer exomes in the last two years has yielded few highly recurrent mutated genes, and none which links robustly to clinical outcome [15], [16], [17]. Although the disparate nature of our cohort prevented systematic discovery of cancer-specific or prognostic editing events (e.g. similar to *AZIN1* in hepatocellular carcinoma [8]), over 500 genes had RDDs predicted to result in deleterious amino acid substitutions, a large proportion of which were recurrent. Interestingly, a recent study of copy number alterations in 125 localized prostate tumors identified a recurrent amplification of region of chromosome 1q22.3, spanning the *ADAR* gene, that was significantly associated with early prostate cancer specific mortality [49]. Clearly therefore, future studies of large prostate tumor cohorts (with appropriate sequencing depth and matched benign) are urgently warranted to evaluate RNA editing as a mutational and regulatory mechanism. Given the inherent flexibility of RNA editing it may be particularly pertinent to identify RDDs in the context of the epithelial plasticity frequently observed in prostate tumors exposed to hormone therapy.

In conclusion, the characterization of RNA editing from RNA sequence data is an emerging field, rife with complexities and controversy. We present a detailed characterization of the landscape of RNA editing in low coverage sequence data from 16 prostate tumor specimens, using a systematic approach which is likely to be applicable to other studies. Our data demonstrates further evidence for RNA editing as an important regulatory mechanism and suggest that the RNA editome be further studied in cancer.

## Methods

### Samples and sequencing

We assembled matched whole-genome sequencing (DNA-seq) and transcriptome sequencing (RNA-seq) data from three previous studies performed in our laboratory. This data spanned 10 prostatic tumors from primary and metastatic sites of 6 patients [50], [51], [52], 2 cell lines (LNCaP and C42) [50], and 2 patient derived xenograft tumors from 1 patient [53], [54]. We added to this dataset a further patient-derived xenograft tumor from another patient [54]. Sequencing was performed at BCCA Michael Smith Genome Sciences Centre according to standard protocols that have been described in [55]. Detailed information is provided in Table S1 in File S2. Data is available at ftp://guest:guest@ftp.prostatecentre.com/RNA-editing/. For additional validation of RNA editing events, we assembled an independent set of prostate cancer samples, which were collected from patients undergoing radical prostatectomy and snap frozen according to the current Vancouver General Hospital pathology protocol. All patients signed a formal consent form approved by the ethics board. For DNA isolation, digestion of 100 µm snap-frozen tumour tissue with 0.2 mg/ml Proteinase K (Roche) in digestion buffer (50 mM NaCl, 10 mM Tris-HCl (pH 8.3), 1 mM EDTA and 0.5% SDS) was carried out overnight at 55°C. Samples were incubated with RNase solution at 37°C for 30 minutes and treated with protein precipitation solution followed by isopropanol precipitation of the DNA. The DNA was further purified by Phenol:Chloroform:Isoamyl Alcohol (25∶24∶1), and precipitated by adding 1/10th volume of 3M sodium acetate and 2.5 volumes of 100% ethanol, before re-suspension in TE. RNA from snap-frozen tissue was isolated using the mirVana Isolation Kit from Ambion (AM 1560).

### Mapping and data processing

DNA-seq reads were aligned onto the human reference genome (hg19/GRCh37) using BWA (0.5.9-r16) [56] allowing 1nt mismatch at most in a 24nt seed. For RNA-seq, reads were mapped onto the hg19 genome and exon-exon junctions by splice-aware aligner Tophat (v1.4.1) [57], using the known gene model annotation from Ensembl release 62. Reads with an unmapped mate or multi-mapped location were filtered out using Bamtools (1.0.2) [58] and PCR or sequencing optical duplicates were marked and removed by Picard (1.55) (http://picard.sourceforge.net). Using NCBI dbSNP build 132, multiple sequence local realignment around InDels and base quality recalibration was performed by GATK (1.4) (The Genome Analysis Toolkit) [59] to correct likely misalignments. Integrating DNA/RNA sequencing data of all specimens, SNVs/InDels were identified and filtered by GATK [60] to achieve high-confidence sites (strand bias, base quality, mapping quality and position bias were taken into account). Additionally for RNA-seq data, we used samtools (0.1.18) [61] to call SNVs/InDels, and retained as high-confidence only those sites which were concordant between both GATK and samtools results. All variants were annotated with genic regions and potential consequences on protein-coding sequences using the tool AnnoVar [62]. The effect of non-synonymous SNVs on protein function was assessed using Condel [63], a method which integrates several predictive tools (e.g. SIFT, Polyphen2, MutationAssessor).

Based on the alignment of RNA-seq reads, gene expression profiles for each sample were calculated based on the gene annotation (Ensembl release 62). Only reads which were unique to one gene and exactly corresponded to gene structure were assigned to the corresponding genes. Raw read counts were normalized by R package DESeq (1.10.1) [64], which was designed for gene expression analysis of RNA-seq data across all samples (Table S17 in File S2).

### The identification of RNA-DNA differences

For each tumor specimen, genotypes of all sequenced DNA and corresponding RNA sites were compared, and separated into 2 categories, either concordant sites (where the DNA genotype matched that of the RNA) or discordant sites (where the DNA and RNA genotype was different).

1. For concordant sites we further divided into 3 subcategories by genotype: homozygous reference (*AA*), heterozygous (*AB*) and homozygous variant (*BB*). For homozygous reference we calculated the average counts of reference DNA reads on all sites (*C_DNA, AA_*). For the 2 other subcategories we calculated the average counts of variant RNA reads (*C_RNA, AB_*, *C_RNA, BB_*).
2. For discordant sites, the following criteria were used to determine high coverage RDD sites:
  - Homozygous reference in DNA (*AA*) and reference read counts *> C_DNA, AA_* (described above);
  - Non-Homozygous reference in RNA (*AB* or *BB*) and variant read counts *> C_RNA, AB_* or *C_RNA, BB_*;

Because our DNA-seq coverage was relatively low (∼4X), we were aware that utilization of the above method alone would miss many potential RDD (RNA-DNA difference) sites, and as such we employed the following method to rescue low coverage RDD sites. For each discordant site with a homozygous reference on the DNA (under the assumption that every site was edited), we integrated RNA genotyping results from all specimens and calculated the average editing ratio: *r = e/t*, where *e* is the sum of edited reads in all specimens and *t* is the sum of covered reads in all specimens.

Then, for each individual specimen, we used a binomial test based on the editing ratio *r* (the average chance of seeing edited reads) to determine whether low coverage was the reason for omission of this site.

1. On DNA level, with *x* reference reads covering this site, the probability of obtaining this number of non-edited reads is *(1-r)^x^*;
2. On RNA level, with *y* edited reads covering this site, the probability of obtaining this number of edited reads is *r^y^*.
3. If *r^y^* and *(1-r)^x^* is both less than 0.05 and *y >3*, which means the random error probability is less than 0.05 on both DNA and RNA, we consider this site as a low coverage RDD site.

### Stringent filtering of RDD candidates

To minimize false positives we applied the following filters:

1. To rule out the possibility that RDD sites could be genuine polymorphic sites or mutations we excluded variants present in dbSNP build 132 (except SNPs with molecular type “cDNA”), which includes variants from the 1000 Genome Project. Given the potential for SNPs not present in dbSNP and the low coverage of our DNA-seq, we rigorously removed sites which were observed in any DNA-seq reads of any specimen. Additionally, we downloaded the COSMIC database [65] and filtered out any sites previously reported as mutations.
2. To exclude false positives resulting from poor mapping quality around splicing sites, we filtered out all sites located within 8bp intronic flanking region of all splicing sites.
3. To exclude potential contamination from the mouse genome [66], we retrieved 61bp of the genomic sequences flanking RDD sites and substituted the RDD site with the edited base. Then we applied BLAT(V3.4) [67] alignment against mouse genome (MM10). If the substituted flanking sequence had a better hit on MM10 than the original flanking sequence and the hit covered the RDD site with identity greater than 90%, then we excluded the corresponding RDD site as potential contamination.
4. To exclude false positives due to mismapping reads from paralogous genes or repeat regions, we retrieved 61 and 101bp of flanking genomic sequences and substituted all covered RDD sites with edited bases, then aligned them onto human genome (hg19/GRCh37) by BLAT. If the substituted flanking sequence was able to be aligned better or equally well on other regions in genome, we discarded those RDD sites as potential false positives.

### Functional annotation of RDD sites

All RDD sites were annotated by genic regions according to Ensembl release 62 (see Table S18 in File S2 for all RDD sites) and illustrated using Circos (http://mkweb.bcgsc.ca/circos). We defined recurrent RDDs as those present in at least 2 samples, and clusters of RDDs were defined as consecutive RDD sites within a 50bp distance or at least 3 RDD sites within a 100bp window. The DARNED database for hg19, which contains 40,485 A→G and 3 C→T RNA editing sites collected from human ESTs studies, was downloaded from http://darned.ucc.ie/download. Conserved elements were predicted using the phastCons algorithm [27], where elements are derived from comparative genome sequence alignment of 46 species. miRNA target regions were predicted by miRanda (downloaded from http://www.microrna.org), and only predictions with a good score were retained. To evaluate transcript expression with and without RDDs affecting miRNA target regions we did the following. A matrix *C(i,k)* was created to store the number of miRNA target RDDs in gene *k* in sample *i*. Then we assigned gene expression data to miRNA target RDDs genes from the above matrix and produced a new matrix *C'(i,k)* storing RDD counts and expression values in nodes for 1,196 genes in 16 samples. Expression data of 135 (10.12%) genes could not be assigned because of different version of gene annotation between miRanda and Ensembl release 62 (Table S12 in File S2). To estimate whether miRNA target RDDs affected gene expression, we classified samples into two groups: with and without RDDs. To minimize the bias from gene expression on detection of RDDs, we considered only genes with approximate (RNA) sequencing coverage greater than 10X. Furthermore, we only evaluated protein coding genes since our negative control was RDDs affecting coding regions. Genes with both miRNA target RDDs and coding regions RDDs were removed from comparisons. Network analysis was performed using Ingenuity (IPA) Knowledge Base 9 (Ingenuity Systems, www.ingenuity.com).

### Validation of RDD sites by Sanger sequencing

To validate RDDs, we amplified the RDD site by PCR from both genomic DNA and cDNA using standard techniques (for primers, see Table S11 in File S2). All amplification products were sequenced using ABI PRISM 310 Genetic Analyzer with standard techniques to confirm identity.

### Data access

Sequence data is available at ftp://guest:guest@ftp.prostatecentre.com/RNA-editing/.

## Supporting Information

File S1 **Supplemental figures S1-S5. Figure S1**, Comparison of down-sampled DNA/RNA-seq data size and detected RDDs number between whole dataset and down-sampled subsets. **Figure S2**, The distribution of RDD site type across different regions in the genome. **Figure S3**, RDD site type distribution in Alu repeat, non-Alu repeat and non-repeat regions. **Figure S4**, The distribution of RDDs in genic regions. **Figure S5**, Percentage of the genes with miRNA target RDDs that were up-regulated in primary prostate tumor in published microarray datasets.
(PDF)

File S2 **Supplemental tables S1-S18. Table S1**, Overview of sequencing libraries. **Table S2**, The distribution of the type of RDDs across different regions of the genome. **Table S3**, RDD site type distribution in different genomic regions. Correlation coefficients among samples for each genomic feature are provided. **Table S4**, RDD sites identified in our study which overlap with the DARNED database. **Table S5**, Comparison of RDDs frequency between conserved elements (phastConsElements46way) and transcript regions. **Table S6**, Type distribution of clustered RDDs. **Table S7**, Overview of total RDDs in different genomic features in each sample. **Table S8**, Genes with RDDs affecting exonic regions. **Table S9**, Rank of gene expression or RDD frequency correlation between pairs of samples. **Table S10**, Deleterious non-synonymous RDDs. **Table S11**, Genes with miRNA target RDDs and their expression in each sample. **Table S12**, Breakdown of miRNA targets with RDDs in each sample. **Table S13**, Correlation between miRNA target RDDs and gene expression. **Table S14**, Validation of mirT RDDs. **Table S15**, Additional vaidation in an independent prostate cancer patient cohort. **Table S16**, Percentage of the genes with miRNA target RDDs that were upregulated in primary prostate tumor from other microArray datasets. **Table S17**, Normalized gene expression levels for each sample. **Table S18**, All identified RDDs.
(XLSX)
