# Supplementary material for: Systematic Identification and Characterization of RNA Editing in Prostate Tumors
Source: PLoS One. 2014 Jul 18;9(7):e101431. doi: 10.1371/journal.pone.0101431 (PMC4103770; doi:10.1371/journal.pone.0101431)

File S1: Figure S1-5

Figure S1: Comparison of down-sampled DNA/RNA-seq data size and detected RDDs number between whole dataset and down-sampled subsets.

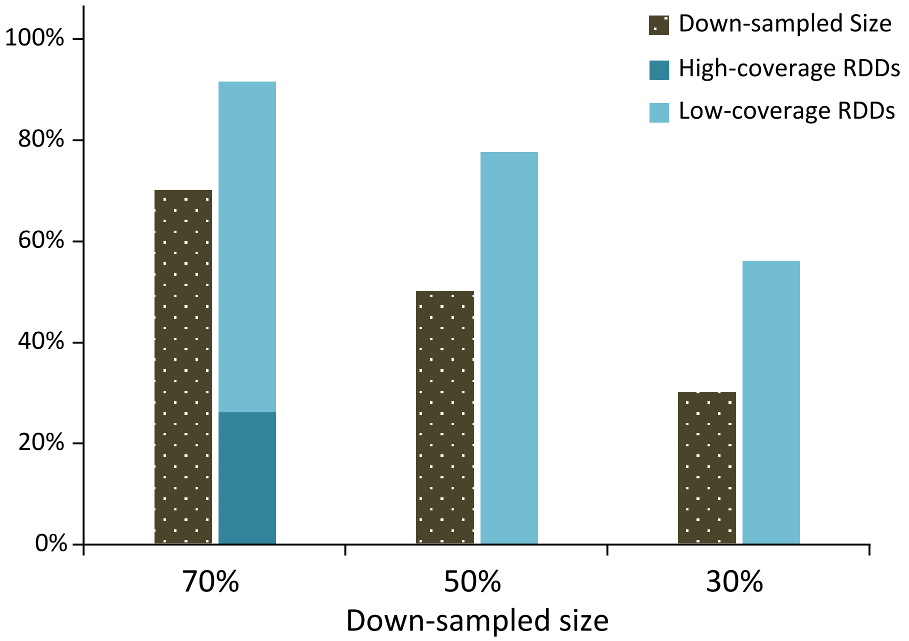

Figure S2: The distribution of RDD site type across different regions in the genome

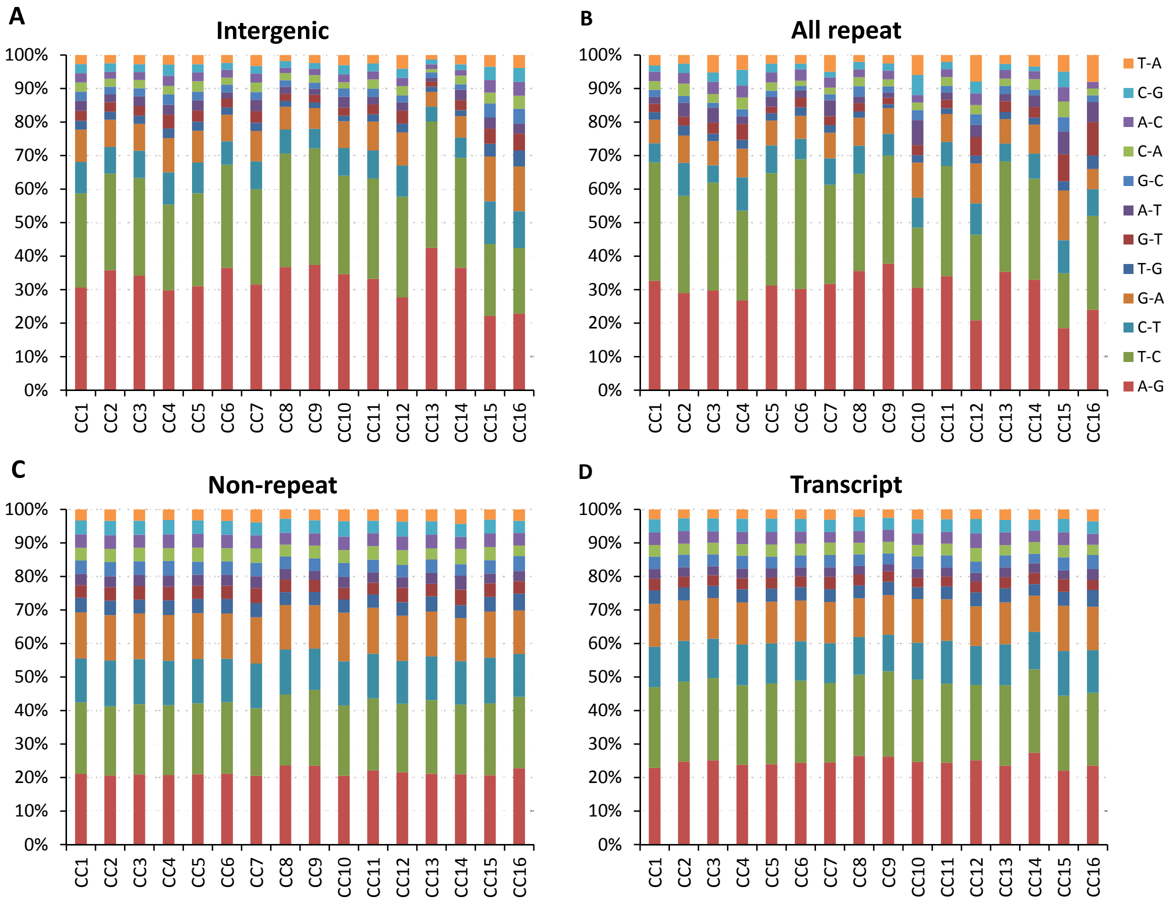

Figure S3: RDD site type distribution in Alu repeat, non-Alu repeat and non-repeat regions

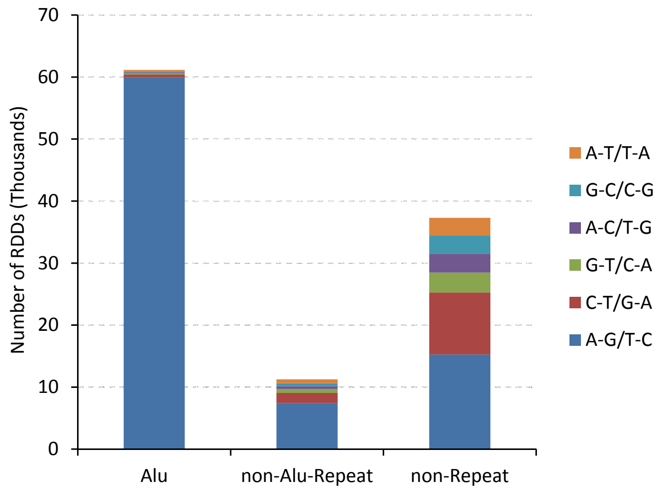

Figure S4: The distribution of RDDs in genic regions. The ratio of intronic RDDs ranges from 8.45% to 61.09% while ncRNAs' ranges from 13.9% to 68.55% and correlation coefficient is -0.95.

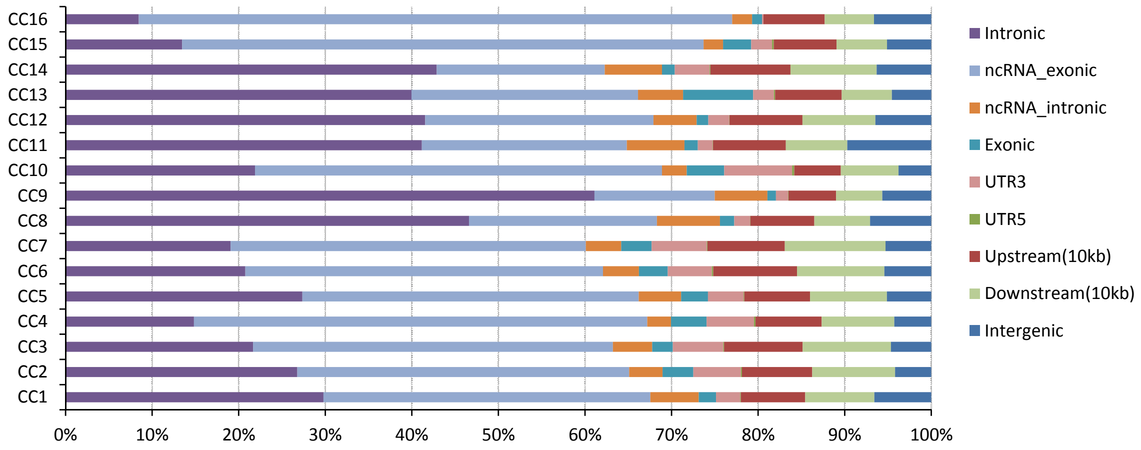

Figure S5: percentage of the genes with miRNA target RDDs that were up-regulated in primary prostate tumor in published microarray datasets.

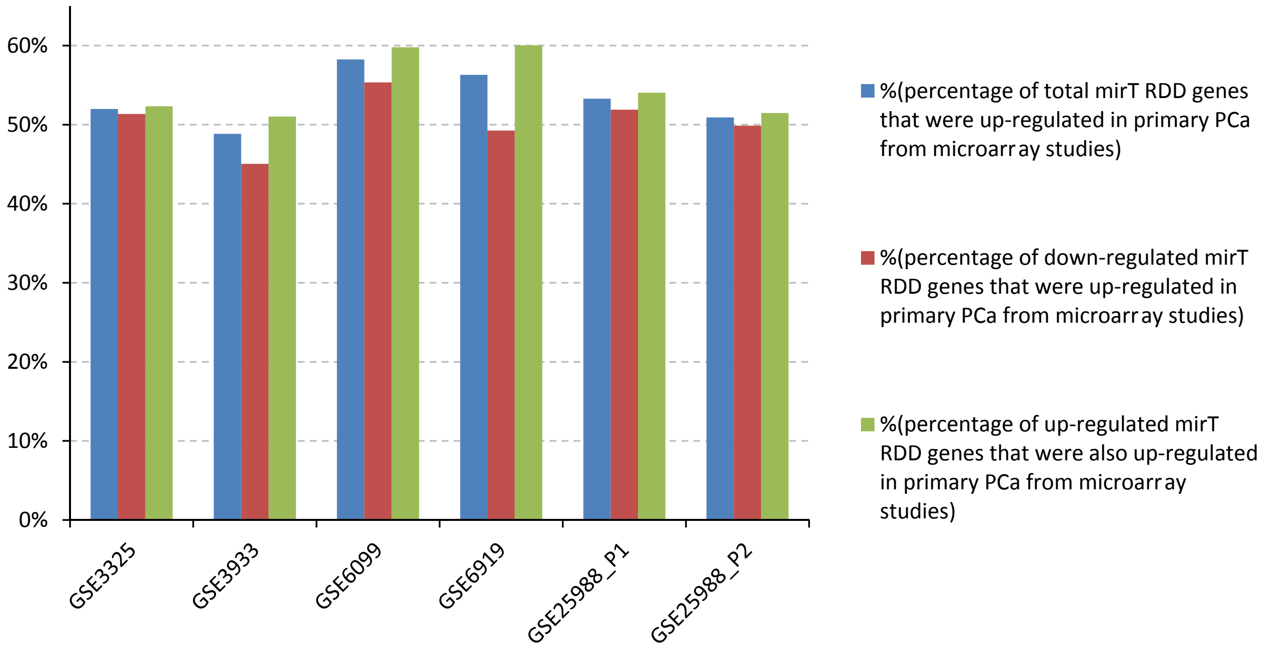

Supplement: File S1 — Supplemental figures S1-S5. Figure S1, Comparison of down-sampled DNA/RNA-seq data size and detected RDDs number between whole dataset and down-sampled subsets. Figure S2, The distribution of RDD site type across different regions in the genome. Figure S3, RDD site type distribution in Alu repeat, non-Alu repeat and non-repeat regions. Figure S4, The distribution of RDDs in genic regions. Figure S5, Percentage of the genes with miRNA target RDDs that were up-regulated in primary prostate tumor in published microarray datasets. (PDF) [file pone.0101431.s001.pdf]
